# Vitamin D receptor and binding protein polymorphisms in women with polycystic ovary syndrome: a case control study

**DOI:** 10.1186/s12902-019-0477-x

**Published:** 2019-12-23

**Authors:** Do Kyeong Song, Hyejin Lee, Young Sun Hong, Yeon-Ah Sung

**Affiliations:** Department of Internal Medicine, Ewha Womans University School of Medicine, 25 Magokdong-ro 2-gil, Gangseo-gu, Seoul, 07804 South Korea

**Keywords:** Vitamin D receptor, Vitamin D binding protein, Polycystic ovary syndrome, Hyperandrogenism, Insulin resistance

## Abstract

**Background:**

Polycystic ovary syndrome (PCOS) is the most common endocrinopathy in women of reproductive age, characterized by hyperandrogenism, oligomenorrhea, polycystic ovary morphology, and insulin resistance. Vitamin D deficiency and vitamin D receptor (VDR)/vitamin D binding protein (VDBP) gene variants could play an important role in susceptibility to PCOS and contribute to metabolic disturbances and menstrual dysfunction. We aimed to investigate the associations of VDR gene and VDBP gene polymorphisms with PCOS susceptibility and to elucidate the impacts of these polymorphisms on the hormonal and metabolic parameters of PCOS.

**Methods:**

We recruited 432 women with PCOS and 927 controls. Polymorphisms in the VDR gene (VDR Fok-I, Cdx2, Apa-I, and Bsm-I) and VDBP gene (VDBP rs4588, rs7041, and rs22822679) were genotyped. A 75-g oral glucose tolerance test was performed.

**Results:**

The distributions of genotypes and allele frequencies in VDR and VDBP genes did not differ between PCOS and control. In women with PCOS, compared to the VDR Fok-I GG genotype, the VDR Fok-I AG genotype was significantly associated with increased levels of total testosterone (β = 5.537, *P* = 0.005). Compared to the VDR Cdx2 AC genotype, the VDR Cdx2 CC genotype was associated with increased levels of fasting insulin and HOMA-IR in women with PCOS, however, the associations were not statistically significant.

**Conclusions:**

This finding indicates that genetic variations in VDR and VDBP were not associated with increased risk for PCOS. In contrast, the VDR Fok-I polymorphism was associated with testosterone level and the Cdx2 polymorphism with insulin sensitivity in PCOS. However, the Cdx2 polymorphism was not significantly associated with increased insulin and insulin sensitivity in women with PCOS after multiple linear regression.

## Background

Polycystic ovary syndrome (PCOS) is the most common endocrinopathy in women of reproductive age, characterized by three fundamental criteria: clinical and/or biochemical hyperandrogenism, oligo/amenorrhea, and polycystic ovary morphology, among which women suspected for PCOS need to present two out of these three criteria for the diagnosis of PCOS, and also insulin resistance (IR) and compensatory hyperinsulinemia [1]. Although several genetic and environmental factors are known to contribute to the development of PCOS, the pathogenesis of PCOS is not clear [2]. Previous studies have suggested that low levels of vitamin D are associated with features of metabolic syndrome and IR in women with PCOS [3–5]. Indeed, causality relationship between vitamin D and metabolic effects has not been established, and longitudinal studies to evaluate the metabolic effects of vitamin D increase in vitamin D deficient PCOS women are yet to be conducted. Serum vitamin D levels were also associated with obesity in women with PCOS, as serum vitamin D levels were significantly lower in obese women with PCOS than in nonobese women with PCOS [6]. Capacity of fat storages of vitamin D may be a potential reason for these associations.

Vitamin D binds to vitamin D receptor (VDR), leading to the transcription of vitamin D-related genes [7]. VDR polymorphisms are known to be associated with metabolic and endocrine parameters of PCOS, including IR and hyperandrogenism [8, 9]. Previous studies have suggested that VDR gene variants were also associated with susceptibility to PCOS [10–12] and the severity of the PCOS phenotype [13]. However, the role of VDR gene variants in the development of PCOS is inconsistent, and the results vary between populations [14], because the VDR gene is polymorphic, and genotype/allele frequencies are variable among different races [15].

The vitamin D binding protein (VDBP) also plays a key role in vitamin D metabolism and is the major protein involved in vitamin D transport [16]. Recently, VDBP gene polymorphisms were associated with vitamin D status in Indian women with PCOS [17]. Moreover, a previous study demonstrated that VDBP gene variants were associated with metabolic syndrome in women with PCOS [18]. However, few studies have examined the associations of the VDBP gene variants with PCOS susceptibility and PCOS phenotypes. Furthermore, no study of the association of VDR gene variants and their role in susceptibility to PCOS has yet been conducted in Korea. The aim of the present study was to investigate the associations of the VDR gene and VDBP gene polymorphisms with PCOS susceptibility and to elucidate the impact of these polymorphisms on the hormonal and metabolic parameters of PCOS in Korea.

## Methods

### Subjects

The present study was conducted using data from the “Biospecimen collection from women with PCOS and control of infertility study” project of the Endocrinology Clinics at Ewha Womans University Hospital to evaluate the menstrual health of Korean women under 40 years of age as we previously reported [19]. Volunteers were recruited by newspaper and online advertisements between December 2008 and October 2010. A trained nurse contacted the candidates by telephone to determine whether they were both able and willing to participate in the study. The volunteers had visited our hospital on the morning of the third day of their menstrual period following an overnight fast of at least 8 h. If the participants had amenorrhea, the blood samples were obtained on a random day. Finally, 432 women with PCOS (24 ± 5 yrs) and 927 non-PCOS control women with regular menstrual cycles (27 ± 5 yrs) were recruited.

The diagnosis of PCOS was based on the National Institutes of Health criteria as follows: (1) amenorrhea or oligomenorrhea (fewer than 10 menstrual cycles per year) and (2) clinical or biochemical hyperandrogenism [20]. Clinical hyperandrogenism was defined as the presence of hirsutism with a modified Ferriman-Gallwey score ≥ 3 [21]. Biochemical hyperandrogenemia was defined as a total testosterone level or free testosterone level above the 95th percentile (total testosterone > 67 ng/dl or free testosterone > 0.84 ng/dl) based on the testosterone levels in healthy women with regular menses [21]. Participants with similar clinical presentations, such as those with congenital adrenal hyperplasia, androgen-secreting tumors, and Cushing’s syndrome, were excluded. Control subjects did not have ovulatory dysfunction or hyperandrogenism. If subjects had taken medication (e.g., steroids, oral contraceptives, metformin, or thiazide diuretics) within the past 3 months, they were excluded [22]. Written informed consent was obtained from all the participants when they visited the hospital, and the institutional review board of Ewha Womans University Mokdong Hospital approved this study.

### Measurements

We measured the height and weight of all subjects, and body mass index (BMI) was calculated as weight (kg)/height (m)^2^. We measured waist circumference in a standing position at the point midway between the lower costal margin and the iliac crest. Blood pressure was calculated as the mean of two manual sphygmomanometer readings with the participant in the seated position.

We measured total testosterone levels via the chemiluminescent immunoassay method using a commercially available kit (Siemens, NY, USA; the mean inter- and intraassay CVs were 4.4 and 6.2%, respectively) which was highly imprecise compared to tandem mass spectrometry. Sex hormone-binding globulin (SHBG) levels were measured by immunoradiometric assay using a commercially available kit (DPC, Los Angeles, CA, USA; the mean inter- and intraassay CVs were 7.9 and 5.3%, respectively). We calculated free testosterone levels using the formula available on the International Society for Study of the Aging Male website, which is based on the total testosterone, SHBG, and albumin levels in single samples from each subject [23]. The free androgen index was calculated as testosterone (in nanomoles per liter)/SHBG (in nanomoles per liter) × 100.

We performed the 75-g oral glucose tolerance test (OGTT) in the morning after an overnight fast. Venous blood samples were drawn at baseline and 120 min after the administration of the 75-g glucose load. Plasma glucose levels were measured via the glucose oxidase method (Beckman Model Glucose Analyzer 2, CA, USA), and insulin levels were measured by radioimmunoassay using a commercially available kit (BioSource, Nivelles, Belgium). The homeostasis model assessment of insulin resistance (HOMA-IR) was calculated as the product of the fasting insulin level (mIU/L) and the fasting glucose level (mmol/L) divided by 22.5 [24].

We genotyped the VDR gene (VDR Fok-I (rs10735810), Cdx2 (rs10875695), Apa-I (rs7967152), and Bsm-I (rs1544410)), and VDBP gene (VDBP rs4588, rs7041, and rs22822679) polymorphisms using a Human Omni1-Quad v1 array. We performed the manual genomic DNA extraction from the stored blood. The success rates for genotyping exceeded 98%, and there was no deviation from Hardy-Weinberg equilibrium (*P* > 0.05).

### Statistical analyses

The statistical analyses were performed using the SPSS 23.0 software package (IBM Corporation, Chicago, IL, USA) for Windows. The Kolmogorov-Smirnov statistic was used to analyze the continuous variables for normality. The levels of fasting insulin, postload 2-h insulin, and HOMA-IR which showed a skewed deviation were logarithmically transformed to achieve a normal distribution. Quantitative variables were provided as the means ± the standard deviations and variables that showed a skewed deviation were provided with medians and interquartile ranges. The between-group differences were assessed using the Student unpaired t-tests and χ2 test, as appropriate. We performed analysis of variance followed by a Bonferroni test for post hoc analysis to evaluate the differences in the metabolic and endocrine parameters between the subjects with different VDR gene variants. Age and BMI were adjusted for analysis of covariance. The associations of total testosterone, fasting insulin, and HOMA-IR with VDR gene polymorphisms in women with PCOS were analyzed with multiple linear regression analyses that was adjusted for age and BMI. *P* values < 0.05 were considered significant.

## Results

Table 1 represents the clinical and biochemical characteristics of the subjects. The women with PCOS were younger than the controls (*P* < 0.05). Body weight, waist circumference, systolic blood pressure, and diastolic blood pressure were higher in the women with PCOS than in the controls (all *Ps* < 0.05). The women with PCOS had higher levels of total testosterone, free testosterone, free androgen index, fasting glucose, postload 2-h glucose, fasting insulin, postload 2-h insulin, and HOMA-IR than did the controls (all *Ps* < 0.05). The levels of SHBG and insulin sensitivity indices were lower in the women with PCOS than in the controls (all *Ps* < 0.05). Hyperandrogenic states and aspects of metabolic syndrome are both commonly associated with lower SHBG levels, which may help explain lower SHBG in PCOS.

**Table 1:** Clinical and biochemical characteristics of the subjects

	PCOS (*n* = 432)	Control (*n* = 927)	*P*-value
Age (years)	24 ± 5	27 ± 5	< 0.001
Body mass index (kg/m^2^)	24.0 ± 4.7	21.1 ± 2.6	< 0.001
Waist circumference (cm)	79 ± 11	73 ± 7	< 0.001
Systolic blood pressure (mmHg)	111 ± 10	107 ± 9	< 0.001
Diastolic blood pressure (mmHg)	72 ± 9	69 ± 8	< 0.001
Sex hormone-binding protein (nmol/l)	45.8 ± 22.8	108.5 ± 62.2	< 0.001
Total testosterone (ng/dl)	76.7 ± 18.6	45.9 ± 15.4	< 0.001
Free testosterone (ng/dl)	1.20 ± 0.43	0.40 ± 0.20	< 0.001
Free androgen index	7.5 ± 4.9	1.9 ± 1.2	< 0.001
Fasting glucose (mg/dL)	87 ± 16	85 ± 7	0.006
Postload 2-h glucose (mg/dL)	112 ± 40	96 ± 19	< 0.001
Fasting insulin (mIU/L)	8.8 (6.3, 12.8)	3.1 (0.4, 6.7)	< 0.001
Postload 2-h insulin (mIU/L)	50.0 (29.9, 88.2)	20.5 (12.1, 35.7)	< 0.001
HOMA-IR	1.83 (1.30, 2.75)	0.63 (0.08, 1.44)	< 0.001

Values are presented as mean ± SD or median (interquartile range)

*PCOS* polycystic ovary syndrome, *HOMA-IR* homeostasis model assessment of insulin resistance

Tables 2 and 3 show the distributions of genotypes of the VDR and VDBP gene polymorphisms in the women with PCOS and the controls. The distribution of genotypes and allele frequencies of the VDR gene (Fok-I (rs10735810), VDR Cdx2 (rs10875695), Apa-I (rs7967152), and Bsm-I (rs1544410)) and VDBP gene (VDBP rs4588, rs7041, and rs22822679) were clearly similar between the women with PCOS and the controls.

**Table 2:** Distribution of genotype frequencies of vitamin D receptor polymorphisms

	PCOS, n (%)	Control, n (%)	*P*-value
rs10735810			
GG	153 (35)	333 (36)	0.675
AG	212 (49)	435 (47)	
AA	67 (16)	159 (17)	
rs10875695			
AC	202 (47)	447 (48)	0.706
CC	160 (37)	322 (35)	
AA	70 (16)	158 (17)	
rs7967152			
CC	28 (6)	46 (5)	0.484
AA	240 (56)	514 (55)	
CA	164 (38)	367 (40)	
re1544410			
GG	386 (89)	826 (89)	0.315
AG	40 (10)	94 (10)	
AA	4 (1)	3 (1)	

*PCOS* polycystic ovary syndrome

**Table 3:** Distribution of genotype frequencies of vitamin D binding protein polymorphisms

	PCOS, n (%)	Control, n (%)	*P*-value
rs4588			
CC	217 (50)	465 (50)	0.945
AC	180 (42)	385 (42)	
AA	33 (8)	76 (8)	
rs7041			
CA	170 (40)	368 (40)	0.992
AA	234 (54)	499 (54)	
CC	28 (6)	60 (6)	
rs22822679			
AA	216 (50)	458 (50)	0.953
CA	179 (41)	392 (42)	
CC	37 (9)	77 (8)	

*PCOS* polycystic ovary syndrome

The associations of genotypes of VDR gene polymorphisms with the hormonal/metabolic parameters of PCOS are shown in Tables 4 and 5. Table 4 shows adjusted means of total testosterone, fasting insulin, and HOMA-IR according to the groups and genotypes of VDR gene polymorphisms after adjustment for age and BMI. The association of VDR Fok-I genotypes and total testosterone was different between women with PCOS and controls after adjustment for age and BMI (*P*-value for interaction = 0.043). In women with PCOS, compared to the VDR Fok-I GG genotype, the VDR Fok-I AG genotype was significantly associated with increased levels of total testosterone after adjustment for age and BMI in multiple linear regression analyses (β = 5.537, *P* = 0.005, Table 5).

**Table 4:** Total testosterone, fasting insulin, and homeostasis model assessment of insulin resistance according to the groups and the genotypes of vitamin D receptor gene polymorphisms

Variables	Groups	VDR polymorphisms	Genotypes	Adjusted mean	95% CI	*P* for group	*P* for genotype	*P* for interaction
TT	Control	rs10735810	GG	45.88	44.09~47.67	< 0.001	0.024	0.043
			AG	46.07	44.51~47.64			
			AA	45.64	43.07~48.22			
	PCOS		GG	73.67	71.01~76.32			
			AG	79.11	76.80~81.42			
			AA	75.51	71.51~79.51			
Fasting insulin^a^	Control	rs10875695	AC	1.41	1.04~1.91	< 0.001	0.087	0.011
			CC	1.06	0.74~1.51			
			AA	3.12	1.77~5.50			
	PCOS		AC	5.74	4.46~7.39			
			CC	7.33	5.51~9.76			
			AA	6.07	3.91~9.41			
HOMA-IR^a^	Control	rs10875695	AC	0.30	0.22~0.41	< 0.001	0.100	0.009
			CC	0.22	0.16~0.32			
			AA	0.66	0.38~1.18			
	PCOS		AC	1.22	0.95~1.57			
			CC	1.55	1.17~2.07			
			AA	1.25	0.81~1.95			

Adjusted for age and body mass index

*VDR* vitamin D receptor, *CI* confidence interval, *TT* total testosterone, *PCOS* polycystic ovary syndrome, *HOMA-IR* homeostasis model assessment of insulin resistance

^a^Logarithmic transformations were applied for analyses and adjusted means are geometric means after adjustment for age and body mass index

**Table 5:** Associations of the genotypes of vitamin D receptor polymorphisms with total testosterone, fasting insulin, and homeostasis model assessment of insulin resistance in women with polycystic ovary syndrome using multiple linear regression analyses

Variables	VDR polymorphisms	Genotypes	β	95% CI	*P*-value
Total testosterone	rs10735810	GG	1		
		AG	5.537	1.679~9.396	0.005
		AA	2.030	−3.312~7.373	0.455
Fasting insulin^a^	rs10875695	AC	1		
		CC	0.255	−0.031~0.542	0.081
		AA	0.056	−0.326~0.437	0.774
HOMA-IR^a^	rs10875695	AC	1		
		CC	0.253	−0.035~0.542	0.085
		AA	0.027	−0.357~0.411	0.890

Adjusted for age and body mass index

*VDR* vitamin D receptor, *CI* confidence interval, *HOMA-IR* homeostasis model assessment of insulin resistance

^a^Logarithmic transformations were applied

The associations of VDR Cdx2 genotypes and fasting insulin or HOMA-IR were different between women with PCOS and controls (*P*-value for interaction = 0.011 and 0.009, respectively, Table 4). Compared to the VDR Cdx2 AC genotype, the VDR Cdx2 CC genotype was associated with increased levels of fasting insulin and HOMA-IR in women with PCOS, however, the associations were not statistically significant in multiple linear regression analyses (*P* = 0.081 and 0.085, respectively, Table 5). The AA polymorphism in controls was associated with higher fasting insulin and HOMA-IR, whereas this specific genotype was not correlated with the same differences in PCOS. It means that the higher insulin and HOMA-IR associated with the AA polymorphism in controls was not found under the presence of PCOS.

## Discussion

These findings indicate that genetic variations in VDR and VDBP are not associated with increased risk for PCOS in our group. In contrast, the VDR Fok-I polymorphism was associated with testosterone levels, and the Cdx2 polymorphism was associated with insulin sensitivity in women with PCOS.

Vitamin D is known to play important roles in the metabolic and endocrine pathways associated with PCOS, such as the insulin signaling pathway [25] and sex hormone production [26]. Several studies have demonstrated that vitamin D deficiency may be correlated with IR, at least in PCOS [3, 27]. Furthermore, vitamin D supplementation has been reported to exert a favorable effect on glucose metabolism in women with PCOS [28]. The exact mechanism involved in the relationship between vitamin D and IR is not clear. Vitamin D is known to affect the expression of the insulin receptor [29] and maintain calcium homeostasis, which is important for insulin secretion by β-cells [30]. Additionally, vitamin D has been suggested to have an effect on the immune system, which is associated with IR [31].

The vitamin D hormone 1,25 dihydroxyvitamin D exerts its functions through binding to the VDR. VDR is a DNA-binding transcription factor and is widely expressed throughout the tissues. When vitamin D binds to VDR, the transcription of vitamin D-regulated genes is initiated. VDR regulates vitamin D levels and calcium metabolism [32]. Additionally, the VDR gene was reported to be associated with IR and insulin secretory capacity [15, 33]. Furthermore, the VDR gene is known to be involved in estrogen biosynthesis and regulation of aromatase gene expression, leading to effects on ovarian function [26]. IR and ovarian hyperandrogenism are key features of PCOS, and thus, VDR gene variants could affect the susceptibility or the phenotype of PCOS.

However, VDR gene variants were not associated with increased risk for PCOS in our group. Gene variants are unlikely to influence the occurrence of PCOS, as variants distributions were similar between PCOS and non-PCOS women. In agreement with the results of our study, the differences in genotypic/allelic frequencies of VDR Fok-I and Bsm-I were not significant between 46 women with PCOS and 46 controls in Iran [34]. In addition, there was no significant difference in genotype and allele frequencies between 181 Iranian women with PCOS and 181 controls with respect to VDR gene polymorphism rs757343 [35]. The CC genotype and C allele of the VDR Taq-I gene polymorphism were significantly more common in 150 Egyptian women with PCOS than in 150 controls [12]. In another study conducted in Iran involving 260 women with PCOS and 221 controls, the distribution of genotypes and alleles of VDR gene variants did not differ between women with PCOS and controls; however, the “A” allele of the rs757343 VDR gene polymorphism was associated with an increased risk of severe PCOS phenotype development [13]. Different sample sizes, differences in dietary intake including that of calcium and vitamin D, and differences in races and environmental factors may affect the differences of these results between the studies. Higher BMI in women with PCOS could influence the activity of certain gene polymorphisms.

In our study, the VDR Cdx2 polymorphism was associated with insulin sensitivity, and Fok-I polymorphism was associated with testosterone levels in women with PCOS, that is, VDR gene variants were associated with the metabolic and endocrine features of PCOS. Previously, the VDR Cdx2 “AA” genotype was reported to be associated with lower fasting insulin and HOMA-IR in 545 Austrian women with PCOS and 145 controls, which was inconsistent with the results of our study [36]. Additionally, the Fok-I genotype “FF” was associated with increased risk for abnormal serum insulin level and IR in women with PCOS in the study of 162 Iranian PCOS women and 162 controls [37]. Furthermore, there have been several studies in which VDR gene variants were associated with sex hormone levels, similar to the results of our study. The VDR Taq-I “CC” genotype was associated with increased levels of LH, and the VDR Bsm-I “GG” genotype was significantly associated with decreased levels of SHBG in 56 Iranian women with PCOS [38]. In the study involving Austrian women, the VDR Apa-I “AA” genotype was associated with lower testosterone levels [36]. The “AA” genotype of the Cdx2 polymorphism showed an association with increased levels of testosterone in Indian women with PCOS and controls [39]. The “CC” genotype of the VDR Apa-I polymorphism was significantly associated with lower androstenedione levels in Indian women with PCOS [40]. Because IR and hyperandrogenism are key features of PCOS, VDR gene variants may contribute to the pathogenesis of PCOS.

VDBP is the major protein involved in the processing of vitamin D and the transport of its metabolites. Up to 90% of the circulating 25-hydroxyvitamin D, the principal circulating vitamin D metabolite, is bound to VDBP [16]. The VDBP gene polymorphisms rs4588 and rs7041 were reported to be strongly associated with low levels of 25-hydroxyvitamin D in 741 white premenopausal women [41]. There were no associations between PCOS and the VDBP gene polymorphisms rs2282679, rs4588, and rs7041 in a study involving 191 women with PCOS and 100 controls from southern Brazil [18]. This result is consistent with the results of our study, in which the distribution of genotypes and allele frequencies of the VDBP rs4588, rs7041, and rs22822679 polymorphisms did not differ between women with PCOS and controls. However, the TT genotype of VDBP polymorphism rs7041 was associated with metabolic syndrome in Brazilian women with PCOS [18], in contrast to the results of our study. Although some studies have shown that VDBP gene polymorphisms were associated with differences in oral glucose tolerance or fasting glucose in nondiabetic populations [42, 43], there is controversy about the association between VDBP gene polymorphisms and the development of diabetes [16, 44]. Further studies are needed to demonstrate the relationship between VDBP gene polymorphisms and the phenotypes of PCOS in various populations.

This is the first study to evaluate the impacts of gene variants involved in vitamin D metabolism on PCOS susceptibility and PCOS phenotype in Korea. However, several limitations in our study to be considered are lack of information regarding vitamin D status, such as dietary vitamin D intake or daily sun exposure. Because we did not measure vitamin D levels, we could not compare vitamin D levels between groups. The lack of serum vitamin D levels is an important limitation, since VDBP levels and its levels of activity may largely depend on their binding status with vitamin D, and different genotypes may be correlated with strength of link with vitamin D, which would eventually lead to differences in metabolic patterns. Also, VDR gene expression also depends indirectly on vitamin D status, which could compromise the consequences of the variants of these genes. Furthermore, we did not measure total testosterone using liquid chromatography mass spectrometry which had been proposed as the preferable assay by an Endocrine Society Position Statement. It is possible that some metabolic abnormalities had not yet been shown in our study because the subjects of our study were younger than those of other studies. Also even being younger, women with PCOS had worse metabolic features than controls. These differences would likely be more prominent if age was similar between groups. Further studies in various age groups are needed to generalize the results. In an adolescent girl, diagnosis of PCOS is suggested basing on the presence of hyperandrogenism in the presence of persistent oligomenorrhea because anovulatory symptoms and polycystic ovarian morphology may be present in normal stages in reproductive maturation. And it is possible that hypothalamic amenorrhea can be classified as PCOS according to the Rotterdam criteria, although it is not common. Therefore, we used the NIH criteria which was not widely accepted for diagnosing PCOS. Differences of criteria for diagnosing PCOS may affect the differences of the results about the associations of the VDR gene and VDBP gene polymorphisms with PCOS susceptibility. Finally, the power of the current study did not reach 80%, which was the least value to identify the differences in the frequency of VDR or VDBP gene polymorphisms between groups. Further studies with larger sample size would be needed.

## Conclusions

In conclusion, VDR gene variants may be involved in the pathogenesis of PCOS via their effects on hyperandrogenism and IR. After multiple linear regression analysis, none between fasting insulin or HOMA-IR remained significantly different according to genotype. Further studies are needed to evaluate whether genotyping of the VDR gene may be useful in young women with PCOS for prediction of IR in large populations.

## Data Availability

The datasets analyzed during the current study are available from the corresponding author.

## Abbreviations

BMI: body mass index
HOMA-IR: homeostasis model assessment of insulin resistance
IR: insulin resistance
OGTT: oral glucose tolerance test
PCOS: polycystic ovary syndrome
SHBG: sex hormone-binding globulin
VDBP: vitamin D binding protein
VDR: vitamin D receptor

## References

[1] Azziz R, Woods KS, Reyna R, Key TJ, Knochenhauer ES, Yildiz BO (2004). The prevalence and features of the polycystic ovary syndrome in an unselected population. J Clin Endocrinol Metab.

[2] Ehrmann DA (2005). Polycystic ovary syndrome. N Engl J Med.

[3] Wehr E, Pilz S, Schweighofer N, Giuliani A, Kopera D, Pieber T (2009). Association of hypovitaminosis D with metabolic disturbances in polycystic ovary syndrome. Eur J Endocrinol.

[4] Krul-Poel Y, Snackey C, Louwers Y, Lips P, Lambalk CB, Laven J (2013). The role of vitamin D in metabolic disturbances in polycystic ovary syndrome: a systematic review. Eur J Endocrinol.

[5] Li HW, Brereton RE, Anderson RA, Wallace AM, Ho CK (2011). Vitamin D deficiency is common and associated with metabolic risk factors in patients with polycystic ovary syndrome. Metabolism.

[6] Yildizhan R, Kurdoglu M, Adali E, Kolusari A, Yildizhan B, Sahin HG (2009). Serum 25-hydroxyvitamin D concentrations in obese and non-obese women with polycystic ovary syndrome. Arch Gynecol Obstet.

[7] Haussler MR, Jurutka PW, Mizwicki M, Norman AW (2011). Vitamin D receptor (VDR)-mediated actions of 1α, 25 (OH) 2vitamin D3: genomic and non-genomic mechanisms. Best Pract Res Clin Endocrinol Metab.

[8] Santos BR, Lecke SB, Spritzer PM (2018). Apa-I polymorphism in VDR gene is related to metabolic syndrome in polycystic ovary syndrome: a cross-sectional study. Reprod Biol Endocrinol.

[9] Han FF, Lv YL, Gong LL, Liu H, Wan ZR, Liu LH (2017). VDR gene variation and insulin resistance related diseases. Lipids Health Dis.

[10] Shi XY, Huang AP, Xie DW, Yu XL (2019). Association of vitamin D receptor gene variants with polycystic ovary syndrome: a meta-analysis. BMC Med Genet.

[11] Siddamalla S, Reddy TV, Govatati S, Erram N, Deenadayal M, Shivaji S (2018). Vitamin D receptor gene polymorphisms and risk of polycystic ovary syndrome in south Indian women. Gynecol Endocrinol.

[12] Amal S, Shalaby SM, Aly NM, Rashad NM, Abdelaziz AM (2013). Genetic variation in the vitamin D receptor gene and vitamin D serum levels in Egyptian women with polycystic ovary syndrome. Mol Biol Rep.

[13] Zadeh-Vakili A, Tehrani FR, Daneshpour MS, Zarkesh M, Saadat N, Azizi F (2013). Genetic polymorphism of vitamin D receptor gene affects the phenotype of PCOS. Gene.

[14] Reis GV, Gontijo NA, Rodrigues KF, Alves MT, Ferreira CN, Gomes KB (2017). Vitamin D receptor polymorphisms and the polycystic ovary syndrome: a systematic review. J Obstet Gynaecol Res.

[15] Ogunkolade BW, Boucher BJ, Prahl JM, Bustin SA, Burrin JM, Noonan K (2002). Vitamin D receptor (VDR) mRNA and VDR protein levels in relation to vitamin D status, insulin secretory capacity, and VDR genotype in Bangladeshi Asians. Diabetes.

[16] Speeckaert M, Huang G, Delanghe JR, Taes YE (2006). Biological and clinical aspects of the vitamin D binding protein (Gc-globulin) and its polymorphism. Clin Chim Acta.

[17] Haldar D, Agrawal N, Patel S, Kambale PR, Arora K, Sharma A (2018). Association of VDBP and CYP2R1 gene polymorphisms with vitamin D status in women with polycystic ovarian syndrome: a north Indian study. Eur J Nutr.

[18] Santos BR, Lecke SB, Spritzer PM (2017). Genetic variant in vitamin D-binding protein is associated with metabolic syndrome and lower 25-hydroxyvitamin D levels in polycystic ovary syndrome: a cross-sectional study. PLoS One.

[19] Song DK, Lee H, Oh J-Y, Hong YS, Sung Y-A (2014). FTO gene variants are associated with PCOS susceptibility and Hyperandrogenemia in young Korean women. Diabetes Metab J.

[20] Zawadzki J, Dunaif A (1992). Diagnostic criteria for polycystic ovary syndrome: towards a rational approach polycystic ovary syndrome.

[21] Escobar-Morreale H.F., Carmina E., Dewailly D., Gambineri A., Kelestimur F., Moghetti P., Pugeat M., Qiao J., Wijeyaratne C.N., Witchel S.F., Norman R.J. (2011). Epidemiology, diagnosis and management of hirsutism: a consensus statement by the Androgen Excess and Polycystic Ovary Syndrome Society. Human Reproduction Update.

[22] Song DK, Sung Y-A, Lee H (2016). The role of serum MicroRNA-6767-5p as a biomarker for the diagnosis of polycystic ovary syndrome. PLoS One.

[23] Vermeulen A, Verdonck L, Kaufman JM (1999). A critical evaluation of simple methods for the estimation of free testosterone in serum. J Clin Endocrinol Metab.

[24] Matthews DR, Hosker JP, Rudenski AS, Naylor BA, Treacher DF, Turner RC (1985). Homeostasis model assessment: insulin resistance and β-cell function from fasting plasma glucose and insulin concentrations in man. Diabetologia.

[25] Łagowska K, Bajerska J, Jamka M (2018). The role of vitamin D oral supplementation in insulin resistance in women with polycystic ovary syndrome: a systematic review and meta-analysis of randomized controlled trials. Nutrients..

[26] Kinuta K, Tanaka H, Moriwake T, Aya K, Kato S, Seino Y (2000). Vitamin D is an important factor in estrogen biosynthesis of both female and male gonads. Endocrinology.

[27] Hahn S, Haselhorst U, Tan S, Quadbeck B, Schmidt M, Roesler S (2006). Low serum 25-hydroxyvitamin D concentrations are associated with insulin resistance and obesity in women with polycystic ovary syndrome. Exp Clin Endocrinol Diabetes.

[28] Seyyed Abootorabi M, Ayremlou P, Behroozi-Lak T, Nourisaeidlou S (2018). The effect of vitamin D supplementation on insulin resistance, visceral fat and adiponectin in vitamin D deficient women with polycystic ovary syndrome: a randomized placebo-controlled trial. Gynecol Endocrinol.

[29] Maestro B, Molero S, Bajo S, Dávila N, Calle C (2002). Transcriptional activation of the human insulin receptor gene by 1, 25-dihydroxyvitamin D3. Cell Biochem Funct.

[30] Pittas AG, Lau J, Hu FB, Dawson-Hughes B (2007). The role of vitamin D and calcium in type 2 diabetes. A systematic review and meta-analysis. J Clin Endocrinol Metab.

[31] Bikle D (2009). Nonclassic actions of vitamin D. J Clin Endocrinol Metab.

[32] James C (2017). Fleet. The role of vitamin D in the endocrinology controlling calcium homeostasis. Mol Cell Endocrinol.

[33] Jain R, von Hurst PR, Stonehouse W, Love DR, Higgins CM, Coad J (2012). Association of vitamin D receptor gene polymorphisms with insulin resistance and response to vitamin D. Metabolism.

[34] Bagheri M, Rad IA, Jazani NH, Nanbakhsh F (2012). Lack of association of vitamin D receptor FokI (rs10735810)(C/T) and BsmI (rs1544410)(a/G) genetic variations with polycystic ovary syndrome risk: a case-control study from Iranian Azeri Turkish women. Maedica (Buchar).

[35] Ranjzad F, Mahmoudi T, Shemirani AI, Mahban A, Nikzamir A, Vahedi M (2012). A common variant in the adiponectin gene and polycystic ovary syndrome risk. Mol Biol Rep.

[36] Wehr E, Trummer O, Giuliani A, Gruber H-J, Pieber TR, Obermayer-Pietsch B (2011). Vitamin D-associated polymorphisms are related to insulin resistance and vitamin D deficiency in polycystic ovary syndrome. Eur J Endocrinol.

[37] Mahmoudi T (2009). Genetic variation in the vitamin D receptor and polycystic ovary syndrome risk. Fertil Steril.

[38] Ranjzad F, Mahban A, Shemirani AI, Mahmoudi T, Vahedi M, Nikzamir A (2011). Influence of gene variants related to calcium homeostasis on biochemical parameters of women with polycystic ovary syndrome. J Assist Reprod Genet.

[39] Dasgupta S, Dutta J, Annamaneni S, Kudugunti N, Battini MR (2015). Association of vitamin D receptor gene polymorphisms with polycystic ovary syndrome among Indian women. Indian J Med Res.

[40] Sur D, Chakravorty R (2015). Genetic polymorphism in the vitamin D receptor gene and 25-hydroxyvitamin D serum levels in east Indian women with polycystic ovary syndrome. J Mol Biomark Diagn.

[41] Sinotte M, Diorio C, Bérubé S, Pollak M, Brisson J (2008). Genetic polymorphisms of the vitamin D binding protein and plasma concentrations of 25-hydroxyvitamin D in premenopausal women. Am J Clin Nutr.

[42] Baier LJ, Dobberfuhl AM, Pratley RE, Hanson RL, Bogardus C (1998). Variations in the vitamin D-binding protein (Gc locus) are associated with oral glucose tolerance in nondiabetic Pima Indians. J Clin Endocrinol Metab.

[43] Iyengar S, Hamman RF, Marshall JA, Majumder PP, Ferrell RE (1989). On the role of vitamin D binding globulin in glucose homeostasis: results from the San Luis Valley diabetes study. Genet Epidemiol.

[44] Ye W-Z, Dubois-Laforgue D, Bellanné-Chantelot C, Timsit J, Velho G (2001). Variations in the vitamin D-binding protein (Gc locus) and risk of type 2 diabetes mellitus in French Caucasians. Metabolism.

